# The narration of status in far-right populist foreign policy: the United States of Trump 2.0

**DOI:** 10.1057/s41268-026-00370-3

**Published:** 2026-02-03

**Authors:** Leslie Erhard Wehner

**Affiliations:** Department of Politics, Languages and International Studies, University of Bath, Bath, UK

**Keywords:** Role theory, Strategic narratives, Status, Populism, United States

## Abstract

This paper focuses on how, during his second mandate, far-right leader Donald Trump tells a story of his nation as having been disrespected in the recent past by national elites and global ones, while the leader and their close circle have the mission to repair that status as part of United States foreign policy (i.e. respect for the status of the US). When narrating a better future, Trump travels to a remote national past to show the possibility of reinstating US stature in the international. While constructing that better future, Trump also starts to unfold a foreign policy story of success to cement the brighter future in a retrospective way given this future has purportedly been previously lived in a more remote national past. Relied on here is symbolic interactionist role theory, strategic narrative analysis and the notion of ‘heartland’ from populism scholarship; this paper also contributes to the study of narratives of roles and populism in the field of foreign policy analysis by engaging with the IR notion of ‘status’. Taking an interpretative analysis approach, this case study shows how far-right leaders like Trump can conceive and play the status or master role of their states in foreign policy via strategic narratives.

## Introduction

Donald Trump’s ‘Make America Great Again’ (MAGA) narration draws on the United States’ past to offer a vision of the future and reinstate the apparent lost stature of this country in the international by prioritising America and American self-interest. Trump has also made constant reference to the historical past of the US as a great nation; most recently, he has rescued the period of President Willian McKinley (1897–1901) as a golden era, as one to emulate and guide the country when reimagining a new future. This is about rescuing the past of a once-grand nation which enjoys the admiration and respect of peer states. Again, in this case the past guides a new future as much as it seeks to prevent alternative damaging ones for the country’s status if not acting now and promptly in the present.

While Trump offers and shares a vision of the future with his audience, he also shows his own subjective views on the status of his country in the international in reference to a lost past and with his messianic figure centre stage in the repairing of such foregone status. Yet, what remains to be analytically unpacked is the way this type of leader constructs and narrates their country’s status as well as how exactly they play it. In other words, the paper seeks to provide some interpretive answers to the following question: How does far-right leader Trump, in the course of his second mandate, construct and narrate his country’s status as part of US foreign policy?

I argue Trump articulates a subjective view of that status to domestic and international audiences alike, ones which have the power to direct the foreign policy of the state. This leader understands status as a narration of needing to reinstate a lost condition in the international. In this narration, Trump offers a vision of a better future in which the promise is to repair the status of the country in the face of the damage caused to it by his predecessors and to prevent other possible catastrophic futures. This story of a nation harmed stresses a sense of the US having been disrespected by national and global elites who have taken advantage of the country or allowed that to happen. Despite always being uncertain, this leader offers a kind of predictable future in his narrations; he can only develop a vision of what lies ahead against a previously glorious past, to reassure followers of the real benefits contained in his vision of the future. This is a mechanism of uncertainty management for Trump’s receptive MAGA audience, as it involves convincing followers time and again of the rightful direction the nation is heading in under him—something which also allows this leader to start showing success (i.e. respect) to be part of the story. Thus, this remote past—narrated as a Belle Epoque of much grandiosity—offers guidance on where immediate predecessors have gone wrong in the performance of status, while making the new vision of the future articulated for the US possible too.

I use symbolic interactionist role theory and strategic narrative analysis in foreign policy (Wehner, 2020), and the notion of ‘heartland’ from populism scholarship (Taggart, 2000, 2004), as cornerstones for the subsequent theoretical elaboration, thus helping to answer the posed research question. The role-theory approach possesses the necessary conceptual apparatus to describe how status can be enacted or played relationally and through the most common social symbol—namely, the use of language (Mead, 1934)—and thus narrations. Only in and through purposeful narratives do roles emerge, and gain meaning and guide action (Wehner, 2020; see also Wehner and Thies, 2014). This prism also offers a dynamic account of how agents understand the social positions of their countries. Role theory also considers both the temporal and spatial dimensions to narratives and roles in which past, present and future interlock: that is, what the father of symbolic interactionism Mead (1932) called an ‘emergent present’. It is in an emergent present that agents seek to manage the uncertainty of a new future by relying on a subjective past as they confront current realities. I understand status as a master role, which is the most salient attribute of an actor (Guimarães, 2020; Thies 2013; Wehner, 2015). A master role can be played or enacted via strategic narrations, and thus experience adjustments according to how the story is being told by the leader in power. In this sense, the understanding of narratives of the master role and of roles in general builds from the notion that narratives are purposeful or strategic. This is due to the fact that they are composed of a set of tools relied on by political actors to amplify their power both domestically and internationally (Miskimmon et al., 2013).

The role conception and role enactment of the master role are key concepts here, along with the subjective gap between both sides. In other words, how your role is conceived in the social world vis-à-vis how it is enacted, played or performed in relation to the prior conception of what it should be. The gap between both sides can be consistent or seamlessly aligned, but the expectation is that Trump will amplify a sense of deviance between role conception (should be) and role enactment (how it is played) in the story he strategically tells of the immediate national past. Role conception can be considered the self-definition of the actor’s own being (ego or the self) and its social position and situation as regards systemic cues and the expectations of other actors (alter or the other) on the role the self should enact (see Harnisch, 2011; Thies, 2010; Wehner, 2025). Role enactment is how an actor plays or performs a social role, which is the actual foreign policy behaviour of ego (Breuning, 2024).

The gap between role conception and role enactment, or how the role has been played in populist foreign policy, is related to the vision of the heartland. This a construction of an idealised world, only becoming possible in reference to an already lived past which makes a projected future real and attainable for the populist leader and their people (Taggart, 2000). Therefore, the story of the heartland allows populist leaders to locate a new vision of the state in the international. In the case of Trump, this future is about vindicating the lost master role of the state, achieved not only by showing how this role has been recently disrespected but also by constructing a new future which exists retrospectively. That is, one stirring respect for the role as previously, realigning and bringing together again the master role conception and role enactment of the country in this new brighter future as a condition which ‘existed’ in the national past. Only the populist leader—as the main character of the story, and with the support of his/her inner circle—is able to achieve this alignment in the narration between role conception and role performance of the master role.

While there is a continuity to the MAGA project and the type of predispositions seen between his first and second presidencies, analysed here specifically is Trump 2.0. In this new mandate, Trump has reduced the size of the state and eroded democratic practice in his country. He has criticised the immediate past (previous governments) while referring to a more remote one as something able to guide and be replicated as part of the new future lying ahead. He talks about where the US should be in the international to his followers, communicating this envisioned future of it being respected once again internationally by peers as well. He has personalised US foreign policy by bypassing its traditional bureaucratic elite (Destradi and Plagemann, 2019). Trump is the representative of a state with the one of the most potent master roles (if not the highest) in the international system: great power. He also claims that the master role of the US has experienced deterioration over time and, accordingly, needs to be swiftly restored. For instance, his recent tariff war with China and the rest of the world contains pieces of the story of the US being disrespected in the past; this policy is framed, then, as helping restore the country’s lost standing (i.e. respect for the master role of the US).

While this paper’s empirical contribution focuses on unpacking the story of the US master role, also theoretical advances are made herewith. From the interpretive venture of analysing foreign policy under Trump 2.0 through the prism of disrespect/respect for the status of the US, also shown is how master roles in foreign policy can be enacted through the type of stories leaders adopt and advance. The novelty lies in the fact that master roles—as synonymous with status—are underpinned by other sets of roles (auxiliary ones) and this is the only way in which the master role can gain meaning and action according to the existing literature (see Guimarães, 2020; Thies 2012, 2013; Wehner, 2015). Thus, the master role or status can be performed and enacted through narratives, thereby acquiring a life of its own independent of auxiliary roles. Further, the following positions itself within the rapidly emerging set of works on populist foreign policy. Here it engages with the key IR concept of ‘status’, in line with recent calls to more readily embrace IR theories and notions (Chryssogelos et al., 2023; Wajner and Giurlando, 2024). Meanwhile, it also brings the notion of heartland (Taggart, 2000) to the populist foreign policy research agenda.

Before turning to the following sections of this article, part of the ‘Special Collection’ on ‘Narratives in Times of Uncertainty’, some caveats are pertinent here. The introduced theoretical framework does not claim to capture status transition or passage from one master role to another, as a process of rising or declining within the international system. It just aspires to unpacking the storytelling of a populist leader about his country’s place in the world and how such narrations of the master role happen. This does not necessarily involve a transition to a different master role, but may simply represent a concern with how to improve the role enactment of the specific status the state already enjoys and for which it is recognised. Improvement in master role enactment is subjective, and it depends on how the leader in question unfolds his/her chosen story of the nation. Further, focus here is solely on the so-called supply side of populism and thus on the leader and their agency within the foreign policy realm.

In the next section, I will engage briefly with the existing literature on populism in International Relations and provide a key definition of the phenomenon emphasising Paul Taggart’s notion of heartland, which will then be incorporated in the subsequent narrative analysis. Here, I will also address the notion of status in IR and show how, as part of the populist leader’s storytelling, it has been overlooked thus far. After, I will present a more thorough discussion of the chosen analytical framework based around the interplay of symbolic interactionist role theory approaches to narratives and status, strategic narratives in foreign policy and the notion of heartland drawn from the populist scholarship. I will also briefly elaborate on the methods used (narrative analysis), as roles are conceived and played within the type of stories which actors in the foreign policy realm choose to cast and advance (Wehner, 2020). Then, I will move to the empirical case of Trump 2.0’s narrations. This will be followed by a conclusion providing some reflections hereon as well as future research possibilities.

## Far-right populism in international politics

Populism has become a vibrant area of study in IR during the last few years, especially since Trump’s first term in office beginning 2017. Scholars have, as such, defined what the key characteristics of a populist foreign policy are as well as the nature of its dynamics in different regions of the world through case studies from Africa, Asia, Europe, Latin America and North America (see e.g. Cadier, 2024; Chryssogelos, 2018; Destradi and Plagemann, 2019; Jenne, 2021; Lacatus et al., 2023; Lacatus, 2023; Lequesne, 2021; Skonieczny, 2018; Stengel et al., 2019; Subotić, 2022; Taş, 2022; Veerbeek and Zaslove, 2017; Wajner, 2021; Wajner and Wehner, 2023; Wehner and Thies, 2021a; Wojczewski, 2023). Some of this research revolves around the process, outputs and style of populism and the populist leader in foreign policy (see e.g. Wajner and Guirlando, 2024), as well as how they have been able to sideline the traditional bureaucratic apparatus of the state in this sector (Destradi and Plagemann, 2019). Far-right populist leaders depict both policymakers and the bureaucracy as part of the undesirable and corrupt elite which has surrendered to globalism and its international makeup and institutions (Chryssogelos, 2018, 2020).

Populism can be conceptualised as a political strategy (Weyland, 2001), as a discursive practice (Laclau, 2005), as thin-centred ideology (Mudde and Rovira Kaltwasser, 2017) and as a political style (Moffitt, 2016). All these notions have a common denominator: the triad of people versus elites and the general will of the people. It is the populist leader who knows best the latter and thus he/she is its true representative, protecting the people from national and global elites. The ‘people’ and the ‘elites’ are manipulable abstract categories, ones which allow the leader to include whoever is deemed to belong to the project, as positioned in contrast to the group or actors excluded from it when casting visions of a new future to come (Wehner and Thies, 2021a). A different approach is that of Taggart (2000, 2004), who talks of ‘the people’ as a derivative consequence of populist leaders’ allegiance and devotion to the ‘heartland’. For Taggart (2000), the people are not the unifying aspect of populism, rather the heartland. The latter is the populist’s vision and construction of an ideal and idealised world which is articulated retrospectively in relation to a lost and better past. In this sense, far-right populist leaders’ performance in the foreign policy realm is driven by what the state is lacking in the present and specifically what it possessed in the past, which in the international politics of populism is ultimately about stature vis-à-vis other actors.

While the scholarship on populism in international politics and populist foreign policy is dynamic and still growing, as mentioned earlier new works have pointed out the need to engage more with IR approaches (Chryssogelos et al., 2023; Wajner and Giurlando, 2024). At the same time, studies on populism in foreign policy have also noted how the systemic implications hereof are not analysed directly by existing research (although see Giurlando et al., 2024). Moreover, most accounts of challenges to the liberal international order do not employ the conceptual toolkit of populist foreign policy (e.g. Ikenberry, 2018; Lake et al., 2021). Yet, new offerings have tried to rectify this blind spot: Barros Leal Farias et al. (2024), for instance, analyse the type of populist order which these leaders’ agency brings to the international system.

Following this call to engage more with IR theorisation in the study of populism in the international, this paper does concern itself with a traditional concept from the IR discipline (status) while simultaneously keeping the leader’s agency centre stage as the main actor revindicating the heartland. It is they who make sense of the social condition of their country through narratives playing out in front of domestic and international audiences alike. Status has been studied as a material condition and as a position synonymous with power by neorealists and others. Here, material capabilities underpin the status position of a country in a peaking order (Gilpin, 1981; Waltz, 1979); such capabilities are key to facilitating transitions within an existing hierarchy (Doran, 1991; Organski, 1958). Volgy et al. (2014) put strong emphasis on military power as a condition for status. Yet, such materiality creates rights and duties for the status holder (usually a great power) vis-à-vis the adoption of a series of different roles and functions. Institutionalist approaches, meanwhile, address how states can achieve or amplify their status by engaging in different international institutions of a multilateral and regional nature (e.g. Alexandroff and Cooper, 2010; Cooper, 2020). Challenging material accounts, Larsson and Shevchenko (2010) take a dynamic approach to status using social identity theory, which stresses the social creativity of agents to manage their identity strategy and social mobility in the international.

Further, Wolf (2011) stresses the importance of respect and disrespect and their effects on the social status of a given actor. Disrespect is understood as non-recognition of that status, while respect concerns how we expect others to treat us—as discernible in an actor’s chosen patterns of behaviour (Wolf, 2011: 112). The author also mentions how respect and disrespect for the status of a given state are always subjective, while having a domestic dimension as well. Yet, how respect and disrespect can be used domestically has not been developed in full thus far. In fact, the domestic dimension of status is an area still to receive much scholarly attention. Götz (2021), when reviewing three important books on status (i.e. Larsson and Shevchenko, 2019; Murray, 2019; Renshon, 2017), mentions that one of the pending tasks in the study hereof is thus closer examination of domestic dynamics and processes. He mentions that ‘some governments may seek to consolidate domestic power by fostering nationalistic pride through the pursuit of international status’ (Götz, 2021: 241). An exception in this regard is the work of Bywaters (2025), who shows how domestic actors engage in status narratives not only to achieve foreign policy goals but domestic ones, too. This is relevant for this work, as Trump adopts a narrative of status at home by indicating to his audience of followers how the country’s international standing has experienced systematic deterioration and hence needs to undergo repair. This status narrative embraces a future which has purportedly been previously experienced in the past, making the populist leader’s vision of what lies ahead an appealing one to the people of the populist project (Taggart, 2000, 2004).

Therefore, the study of status has focused mainly on transitions from one position to another, and on the mobility strategies of actors seeking new standing. However, the mentioned literature does not analyse how status narrations which are international are also grounded in domestic political processes and target particular audiences on the home front accordingly. This is relevant for the study of populist foreign policy, as the leader seeks to consolidate both his/her own position and that of the populist project at home. This is similar to the point made by Homolar and Löfflmann (2021), who show how populist leaders are able to locate the story of the country’s constant humiliation and thus promise to restore a lost sense of greatness to it. Yet, they do not directly engage with the notion of status or what they deem to have been institutionally and symbolically humiliated by others.

Likewise, not much is said in the existing literature about how states or their leaders enact a particular status which has already been achieved and it is recognised by others. In fact, leaders may adopt ‘status narratives’ (cf. Bywaters, 2025) to simply adjust and correct the existing status of their country if there is a sense that this master role was not performed or enacted in the best possible way by their predecessors in the recent past. This is, of course, subjective, as populists and indeed any type of leader can strategically articulate a narrative of disrespect which is also promoted internationally as part of their foreign policy. This also targets the domestic political arena, namely as a means to undermine the scope for action of political adversaries and solidify the bond with followers of the populist project. This is also part of the broader strategic narrative laying out a ‘new’ retrospective vision of the future or the heartland, seeking to achieve the desired remedying of the sense of disrespect present in terms of how the master role has been previously enacted by the state in question.

## Master-role narrative in an emergent present

### Role-theory concepts

Roles are patterns of appropriate behaviour and the type of actor it is possible to be within an organised group (Harnisch, 2011; Thies, 2010). Roles are relational in the sense that other actors (alter) are always present in the process of their conception, namely via articulated counter-roles or the expectations communicated to the self (ego) (Oppermann, 2024; Wehner, 2015). As mentioned, a role conception encompasses self-definition of who you are in a given social context vis-à-vis said cues and demands (Thies, 2010) and role enactment is the actual foreign policy behaviour of the actor (Breuning, 2024). As role conception provides a template of possible courses of action for the self, the enactment of it along with the accompanying expectations of others can feedback into that conception and thus adjust and redefine the role in incremental ways (Wehner, 2025). Unless, that is, the self is exposed to a shock of high magnitude, whereby the role can undergo deeper redefinition (Thies and Wehner, 2023).

As mentioned earlier, the gap between role conception and role enactment will, when amplified by domestic role contestation (Cantir and Kaarbo, 2012, 2016), role conflict induced by contradictory demands from external others (Wehner, 2016) or by the leader’s own agency in tweaking a role of the state (Wehner and Thies, 2021b), tend to create a deviance which can be politically exploited and manipulated via strategic narrative choices. Yet, certain roles are more essential than others for defining who the actor is in their social life. A hierarchy exists within the role set of an actor and as regards the number of different ones they possess (Aggestam, 2006). Thus, the master role, as mentioned, is treated as the actor’s most salient attribute. Thies (2013) talks of master role in a more structural sense, but both Guimaraes (2020) and Wehner (2015) stress the possibility of master roles (always interactional) such as great power, intermediate, regional power and small power existing. This also opens up the possibility that master roles are not static; they are not only infused with meaning and action through auxiliary roles or the types of functions underpinning the master role (such as leader, mediator and so on) (Breuning and Pechenina, 2020; Thies, 2013; Wehner, 2015). Rather, this occurs through own performance as well—namely, via narrations representing the state status in the international.

### Strategic narratives in role theory and the heartland in populism scholarship

Symbolic interactionist role theory in the foreign policy realm has relied on the benefits of narrative analysis (Wehner, 2020), and more broadly on the narrative turn in IR (see e.g. Berenskötter, 2020; Freistein and Groth, 2025; Goddard, 2009; Hagström and Gustafsson, 2019; Jackson, 2006; Krebs, 2015; Miskimmon et al., 2013; Oppermann and Spencer, 2016; Spencer, 2016; Subotić, 2016). Symbolic interactionist role theory has creativity at its core, as agents (states, leaders or foreign policy elites speaking on behalf of the state) can reimagine an existing role by reframing events as well as relationships with others; adjusting or changing the type of story they tell both domestically and internationally are further possibilities. Only through their own narrations can these foreign policy actors make sense of who they are, what they want as well as be able to manage the inherent social novelty faced when enacting roles. Through the stories they tell, actors are able to manage and reduce the anxiety and inherent uncertainty the unknown (the future) poses: ‘Thus, the agency of an actor is also based on how ego reinterprets its past to make sense of current challenges in foreign policy, as well as to frame possible courses of actions in future’ (Wehner, 2020: 364). Carr (1986: 14–15) cited here Paul Ricœur’s hermeneutic approach to narratives, which emphasises how social life is made of storytelling: without narratives, the social world can never take form. Thus, narratives give coherence to actors combining historical realities, as much as fictional myths of the past treated as if they were social truths (Ezzy, 1998).

Symbolic interactionist role theory is narrative-based. Mead (1932) not only developed a social–psychological approach to the self and other, he also situated role interactions within a temporal dimension (Ezzy, 1998). Hence, narratives are a subjective recreation of events in an intersubjective present (Wehner, 2020). Role interactions and enactments—in this case, of the master role—unfold in present time (Simpson, 2014: 8–9). But, for Mead (1932), the present as a temporal dimension is always a passageway to a different future, as actors rely on the recurrent reinterpretation of the past as they tell their stories. These narrations and the constant re-evaluation of the past also serve the purpose of dealing with the uncertainty or unknowns around what lies ahead (see also, Mead, 1963/64).

Mead (1963/64) does not talk of emergent present as a strategic process of storytelling since for him all social actors enjoy this capacity of making new futures as the experience an emergent present. However, in foreign policy, agents or actors are influential subjects who can at times direct and redirect the story in a strategic way and make sense of structural constraints in their stories. Thus, strategic narratives are a good analytical fit for the study of foreign policy roles from a leader centric perspective. Populist leaders as central actors of the story are influential decision-makers able to experience this emergent present and tell stories of (master) roles to achieve a desired goal and create effects in the domain of foreign policy. Roles in foreign policy are part and present in a strategic narrative of the populist leader. In this sense, strategic narratives ‘are a means by which political actors attempt to construct a shared meaning of the past, present and future of international politics to shape the behaviour of domestic and international actors’ (Miskimmon et al., 2017: 6).

Strategic narratives help the populist leader to legitimate his/her set of actions, and mobilise domestic support to reinforce the bond with the people of the populist project as much as to reduce the effects of the counternarratives of other states and national and global elites (adapted from Miskimmon et al., 2015: 57). Yet, such mobilisation, legitimation and bonding occur in relation specifically to the heartland in populist storytelling. The heartland is a manipulable, powerful and at times diffuse and obscure social construction which only becomes meaningful through narratives the populace can identify with (Taggart, 2000: 4). Populists evoke in their stories a specific vision of the heartland which resonates with the followers of the project as the idea of a place they have been before. The heartland contains a reimagined but still predictable future, one in which the people should find themselves. This story of the heartland as going to the future in a retrospective manner makes populist leaders’ vision of what lies ahead desirable to the people of the populist project while also reducing the uncertainty around it. This is different from a ‘utopia’, however: the latter is an idealised project not necessarily connected with the cultural material of the past, while the heartland is consciously constructed backwards via strategic narratives. Within the notion of the heartland there are also exclusions or frontiers as regards corrupt and demonised national and global elites (Taggart, 2000: 96), and in international relations with concern to other states and leaders seen as anathema to the populist mission and project.

Thus, the heartland takes form through the storytelling of the leader, as a narrative entrepreneur (Subotić, 2016). The populist leader, as such, is the causal force who can strategically change both the story and the environment in which they operate. They can exercise control in order to stretch the past, either as a negative or positive pool of ‘storical’ elements, to cast a new story. Yet, not all elements must come together in a coherent way: narratives always have their contradictory elements. It is the narrative entrepreneur who holds the story together despite this dissonance. As this entrepreneur enjoys the agency to articulate and locate a narrative which becomes a key source of political power (Berenskötter, 2020), he/she also experiences contestation and even structural constraints vis-à-vis what types of storytelling are deemed socially acceptable by their captive audience. Not all new narratives are readily embraced, as their resonance depends on their *internarrativity*—that is, the extent to which they can intertwine with pre-existing narrative tropes and shared understandings (Hagström and Gustafsson, 2019). In other words, new narratives need to be recognised as part of the national milieu in the collective memory of loyal followers, despite other groups framed as elites by the leader potentially resisting and/or undermining a particular version of reality. It is here where the notion of heartland is located, as it is the site of the past which the people and the populist leader want to go back to and thus achieve in the future as well.

The heartland in foreign policy unfolds in the populist leader’s articulation and manipulation of a story which shows the existing disrespect for the master role of the state to both domestic and global audiences, with the promise made that the individual in question is there to repair such lost stature by bringing back respect for the status which the country once previously enjoyed. This disrespected master role, as much as the sense of bringing back the desired respect for it, is something which happens in an emergent present: it is here where past, present and future time come together and interlock (Mead, 1932: 73–75). The emergent present is always the product of interpretive processes by agents who, in telling new stories of the past, make novelty emerge (Wehner, 2020).

For the purposes of examining Trump 2.0, agency is also at the heart of each new round of his storytelling about the past in pursuit of making sense of the present by offering a new vision of the future. Creativity around how a master role is conceived and enacted does not always need to be about total disruption, expressed in a radical change in the story being told, but it is about adjusting the role as the narration unfolds. This helps to reinvigorate the state’s master role. The emergent present is about possible pasts as much as it is about possible futures, with potential pathways ahead beginning to become apparent in the here and now. In these narrations, both role conception and role enactment happen within what is being communicated to the external world and domestic followers alike, but especially to the latter in order to manage and reduce uncertainty around what comes next for the nation or the people of the project (versus the elites).

**Fig. 1:**
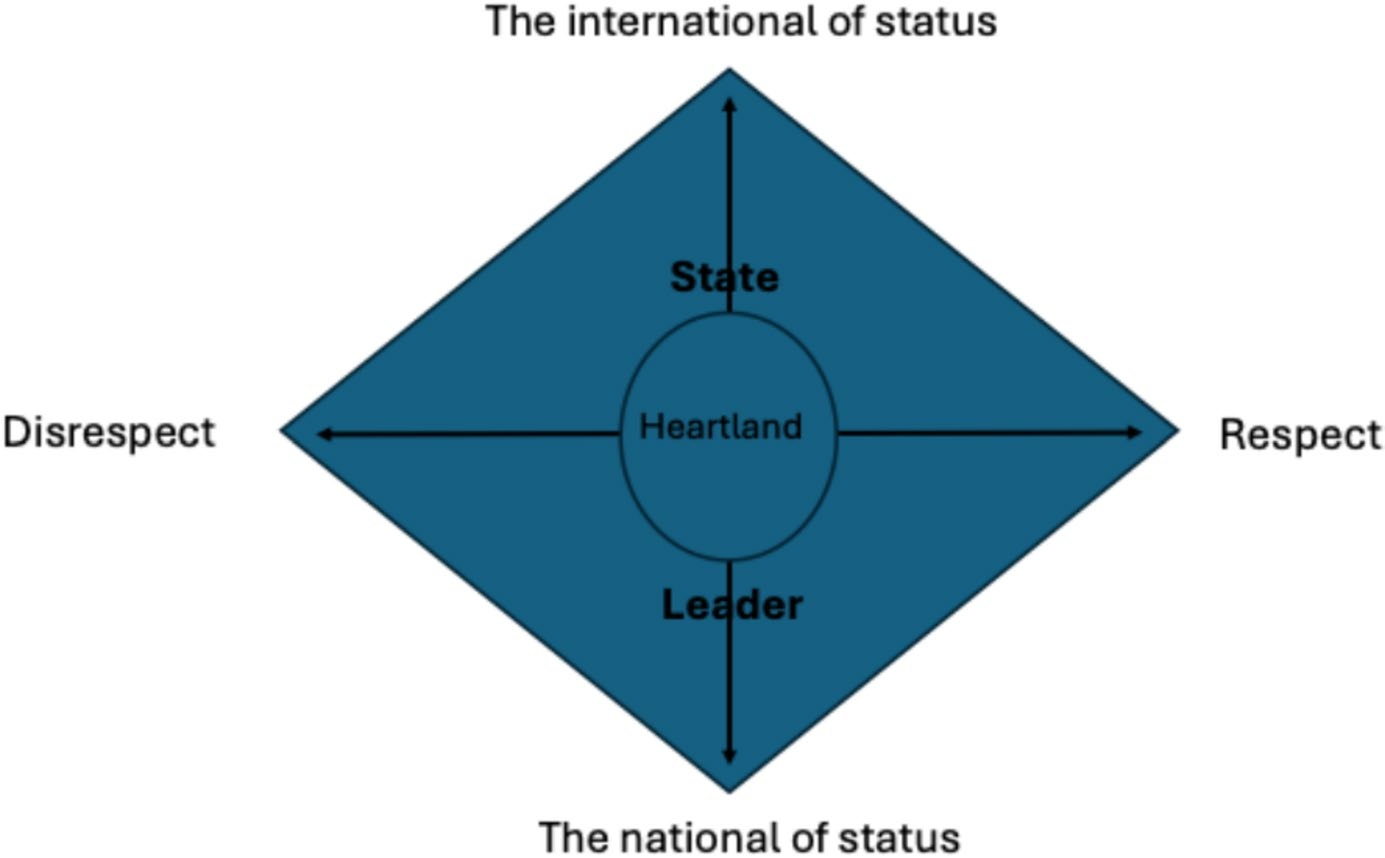
Framework of master-role narratives. *Source* Author’s own compilation

It is expected that the strategic narrative advanced by Trump 2.0, as the new voice of the state, is of a recent past in which the US has been disrespected by elites at home (domestic dimension of status) and abroad (external dimension thereof) in its enactment of the master role. This follows a perceived mismatch between role conception and enactment, which is articulated by and present in the story which the leader delivers. It is here in this gap that the leader as narrative entrepreneur promises a future of full respect being given to the master role of the nation (see Fig. 1). The dichotomy of respect/disrespect as regards the master role is always present in the story; such references are considered part of the status narrative of the populist leader in their quest to bring esteem back to the nation and the heartland.

As much as this past of disrespect for the master role is negative, the story of it is dialectical; the populist leader, as the saviour of that nation, can also rely on other moments from times gone by to show the best way to correct or align subjective views when articulating a vision of the future in an emergent present. These other moments serve the purpose of showing that respect is indeed attainable; any actions taken by the leader to achieve the desired respect once more in this emergent present are deemed feasible. In this manner, the envisioned future becomes acceptable to their followers, while the narrative is also able to sideline and reduce the scope of other rival narrations promoted by elites. Finally, it is the enactment of the master role which provides the necessary theatricality to convince one’s audience of the rightness of the narrative advanced by the leader despite the tensions and contradictions inherent to it.

### Methodological considerations

In a methodological sense, a ‘narrative’ is a spoken or written text which describes and interprets an event or a series of actions connected within some sort of sequence (cf. Czarniawska, 2004: 17). As stressed earlier, examining the deviance between the master role’s conception and enactment, as articulated in the story told, is the preliminary step in the empirical work. First, the populist leader as narrative entrepreneur presents this existing gap to the captive domestic audience. Second, they position a story of being disrespected in contrast to how things were in a more glorious past when the state in question was widely admired by external actors i.e. respect. Third, an emergent present, in which the main storyteller is located, is also included, as a way to make sense of the recent and remote past and to embed the envisioned future. Such choices seek the reinstatement of a positive image of one’s national status among the audience of followers. These aspects are at the core of the story told, which is methodologically analysed along the lines of setting, characterisation and emplotment (cf. Oppermann and Spencer, 2023).

The setting is the location where the play takes place, and it helps to portray key dimensions of the foreign policy decision-making process—such as what it is at stake, the set of relevant issues to be decided on and promoted, as well as whether the issues and decisions narrated are to be represented as challenges and opportunities and/or with a sense of urgency and threat. Characterisation gives the audience clues over the meaning of the story, helping unpack the motives, interests and behaviour of the individual and collective characters. It takes form in the descriptive aspects of the actors involved as much as it does in value judgments around what they do and how they do it (i.e. the way they enact foreign policy). Labelling an actor not only with names and titles but also with the categories of bad/good, bully/calm or hostile/peaceful generates expectations concerning the play’s nature and how a leader or government will interact with other external actors (i.e. relationality). Emplotment is understood as the type of events articulated in sequential order to give a story coherence, establish causal relationships and render this version of reality readily accessible. It also allows the audience to generate their own judgement and evaluation of the situation and story being narrated (cf. Oppermann and Spencer, 2023).

The setting in which the story is narrated is the domestic stage, recreated by looking at how Trump addresses a national audience from the White House, Congress and via social media channels. The domestic is not a closed setting, as it is intertwined with an international stage: what Trump purposefully narrates shapes foreign policy encounters, too. In this case, Trump also replays part of the story of the master role of the US being disrespected and how he is there to remedy that when interacting with China (bilateral setting) and with other Asian and European peers—both bilaterally (with specific states) and multilaterally (with ASEAN or EU leaders).

In our examined case the dominant actor is Trump, who also is the main character telling the story. The characterisation of Trump is of a hero and an actor putting the American people first, which becomes his main mission. Alongside this virtuous character there are antagonists as well. To these the leader attributes responsibility for the deterioration (disrespect) of the state’s master role. For instance, Trump represents Joe Biden as incompetent, the worst US president ever, and as a slow-paced decision-maker who decided to favour the corrupt political national and global elites over the American people. Other characters of the story are the American people, who are represented in a positive way in regard to the national past—depicted as being pioneers, adventurous and full of virtue, while the political elite is characterised as the enemy and nefarious.

International actors such as China and its head of state are characterised in a dual manner. This is also the case with European and Asian states and leaders as well. On the one side, they are depicted as clever, opportunistic and smart for benefitting from the US over the years. They are narrated as taking advantage of the US, as the key feature of it having been purportedly disrespected. On the other side, they are also represented as common-sense actors who will likely accept new US conditions and requests to achieve the lost but desired respect for the master role if they do not want further punitive measures to be imposed. In a more abstract way, other characters are global institutions and the global system, represented as hostile to the populist leader and their core mission.

Emplotment deals with the causal temporality of the story. In this sense, the story to be analysed starts when Trump commences his second term in office as president of the US. This new beginning gives him a window of opportunity to make good on his electoral promises regarding foreign policy issues. At the same time, this new mandate makes Trump return narrative-wise to his previous presidency—thus marking events as both a continuation of his first period in office and as a new opportunity to deliver on his promise of making America great again. In the sphere of foreign policy, Trump’s goal is to reinstate the stature of the US as the most important nation in the world (i.e. respect for the master role), and to stop other countries and institutions taking advantage of it (i.e. disrespect). Only from a position of re-established respect to the master role, the US under Trump can become a leader through strength as envisaged by this leader. The emplotment of the story also suggests that the means to achieve the explicitly mentioned foreign policy goals is to exert direct pressure on each and every country disrespecting the US master role (e.g. retaliatory economic measures or publicly blaming them), while the common means is to impose tariffs on all other countries and/or use an all-encompassing framing of how other countries should follow the US without much room being left to contest Trump’s articulated views and story.

To reconstruct the US master-role narrative gaining currency under Trump 2.0, I rely on relevant primary materials. Different speeches he has given—the inaugural speech, the one delivered in Congress and the one announcing his new tariffs policy—since taking office for a second time are hence examined, as also are Trump’s encounters with China’s Xi Jinping in South Korea, ASEAN leaders in Malaysia and interactions with European leaders such as UK Prime Minister Keir Starmer. While the mentioned events are the main corpus for recreating the story of the master role, I also checked Trump’s Truth Social account in the same time period (i.e. over the course of 2025). On this social media channel, Trump’s messages replicates in a synthetic way key elements of his story told both to the nation and external peers in the mentioned public speeches and press declarations when meeting with other key political figures.

While his utterances on this platform are part of the story, they are not much different in substance from the main aspects of his public speeches. Hence, I only use them as background information to supplement the reconstruction of the story on the basis of the aforementioned speeches and interactions; a single such post is drawn on in the empirical analysis, meanwhile. I searched here for keywords denoting relationality especially, like status, respect, disrespect, humiliation, rip-off, tariffs, tariff war, China, Russia, NATO, Europe, the EU, elites, global elites, Joe Biden, MAGA, Americans, the American people, or the people. Thus, all of the materials mentioned above help capture both the domestic and international dimensions of the master-role narrative. I also draw here on secondary accounts to provide contextualisation as regards his first mandate, as Trump’s new presidency reveals patterns of continuity vis-à-vis his previous term in office.

## Trump 2.0 and the story of reinstating US stature

The latest Trump presidency has brought MAGA ideas back into the foreign policy domain once more. These frames have been present in Trump’s narrative ever since he first entered the political arena as a US presidential candidate in 2015. The appeal of the ‘America First’ and MAGA message lies in the fact they contain elements of recuperating something the nation has lost (Skonieczny, 2018, 2019). Trump also speaks regularly of the people versus the elites, in which the later needed to be fought against for benefitting themselves at the expense of the American worker. The Manichean view of the people versus the elites is a key component of the blame attribution to be found in Trump’s long-term narrative, something which continued once he took power for first time (Löfflmann, 2024).

For too long, a small group in our nation’s capital has reaped the rewards of government while the people have borne the cost. Washington flourished, but the people did not share in its wealth. Politicians prospered, but the jobs left, and the factories closed. The establishment protected itself but not the citizens of our country. (Trump, 2017)

Trump 1.0 (2017–2021) continued narrating a story of ‘Crisis America’ in which he romanticised the past to show his receptive audience at home the need to repair the current state of the nation and vindicate its people anew, thus helping achieve a different future for them (Homolar and Scholz, 2019). This antagonistic story allowed Trump to locate himself in the narration as the saviour and repairer of the lost status or master role of the US. He also included herein the global elites to be found in the ranks of multilateral institutions, leaders of other countries and the figure of economic globalisation at large (Wehner, 2022). Homolar and Löfflmann (2021) refer to a story of fear in which the main character narrating events tells the people of how they have been cheated and abandoned by a political elite which should have taken care of them instead: that is, what these authors call a ‘narrative of humiliation’. In this framing, Trump as the main actor is working to reinstate the dignity of the people who have been humiliated: ‘Let’s not let our great country be laughed at anymore’ (Trump, 2016, quoted in Homolar and Löfflmann, 2021: 4).

This tale of shame is not just about the American worker. It also involves vindicating again the social fabric of what the US is in the eyes of the leader as well as the position of the state in the international—in other words, its master role. In fact, Wolf (2017) talks of the importance Trump attributes to symbolism in foreign policy; more specifically, to US status and how it has been disrespected by others. Trump’s first presidency was about his country being number one again and therewith regaining the respect lost during previous incumbents’ presidencies. Again, such a sense of humiliation and disrespect is not just for the American people but for ‘the collective persona of the United States’ (Wolf, 2017: 103). During his first presidency, in some speeches Trump talked about his mission of regaining lost respect and the fact that the US as an international actor had been taken for granted (Lacatus, 2020). ‘America is winning again, and America is being respected again, maybe respected like never before, because we are finally putting America first’ (Trump, 2018). In his farewell speech on leaving office, he stressed that he had reinstated the desired respect for the US’s master role: ‘We restored American strength at home and American leadership abroad. The world respects us again. Please don’t lose that respect’ (Trump, 2021).

Trump 2.0, meanwhile, demonstrates a pattern of continuity here, as he has focused once more on reinvigorating the strength of the US and reinstating a lost national past. While he achieved a sense of respect from others under his previous presidency, Trump claims in his latest narrative that respect for the stature of the US was lost again during Joe Biden’s tenure. Trump positions a gap between the master role conception and role enactment under his immediate predecessor. In fact, it is only Trump who can vindicate anew the US’s master role. In this story, then, Biden becomes the new antagonist or reference point in seeking to locate the repairing of lost status: that is, closing the perceived significant gap which exists between role conception and enactment.

The Biden administration is deemed the incarnation and source of all the problems manifesting in current circumstances seeing the abandonment of the people by the country’s political elite: ‘As we gather today, our government confronts a crisis of trust. For many years, a radical and corrupt establishment has extracted power and wealth from our citizens while the pillars of our society lay broken and seemingly in complete disrepair’ (Trump, 2025a). Further, Trump also says about Biden’s legacy: ‘This country was heading for a collapse under the people that you saw. They were horrible. I think one of the reasons people like the job -- I had my highest approval ratings because I think they’re comparing me to the worst administration in the history of our country’ (Trump, 2025c). And, in more simple terms, he states: ‘In comparison, under Joe Biden, the worst president in American history’ (Trump, 2025b). In a general tone, when narrating his new tariffs policy, he recounts how: ‘I don’t blame these other countries at all for this calamity. I blame former presidents and past leaders who weren’t doing their job’ (Trump, 2025c).

As the narration takes place in an emergent present seeing all three time dimensions interlock, Trump 2.0 not only adopts an antagonistic tone on the country’s most recent past (previous government) as a reality of the present and as an undesirable potential future, but he also relies on the cultural material of the remote national past to construct and provide a new vision of what lies ahead. Desirable futures become more tangible herewith, and thus provide a degree of certainty to one’s audience if they build from the country’s existing cultural repertoire—in this case, the heartland. The remote past is cherry-picked and thus stretched and manipulated in the populist leader’s presented narrative, as a means of reinstating respect for the master role. Sometimes the story is specific to a particular historic figure or role model to be emulated in the present time, on other occasions references to the remote past are more generic ones about the strength of the country and its people. Reinstating the lost respect for the master role is possible and achievable only on the basis of that glorious past, as rendering the populist leader’s political project acceptable to their captive audience.

Trump 2.0 has also talked of President McKinley as a wealth generator for the country through his own tariffs policy during his address to Congress and on social media. ‘[W]e will restore the name of a great president, William McKinley, to Mount McKinley, where it should be and where it belongs’ (Trump, 2025a). He also stated regarding the new tariffs policy, in reference here also to President McKinley once more, that: ‘From 1789 to 1913, we were a tariff-backed nation, and the United States was proportionately the wealthiest it has ever been’ (Trump, 2025c). On other occasions, Trump provides a more general story which resonates with those seeking reassurance that a new destiny awaits the country in the years ahead. By going to the past, the populist leader is able to manage the uncertainty of the future:

Our ancestors crossed a vast ocean, strode into the unknown wilderness, and carved their fortunes from the rock and soil of a perilous and very dangerous frontier. They chased our destiny across a boundless continent. They built the railroads, laid the highways, and graced the world with American marvels, like the Empire State Building, the mighty Hoover Dam, and the towering Golden Gate Bridge. (Trump, 2025b)

In most of his speeches since taking office for a second time, Trump has referred to the past grandiosity of the US and its Belle Epoque: ‘There’s no nation like our nation […]. Americans are explorers, builders, innovators, entrepreneurs, and pioneers. The spirit of the frontier is written into our hearts. The call of the next great adventure resounds from within our souls [...]’ (Trump, 2025a).

Hence, a strategic narrative as advanced by a leader without a vision of the future is always incomplete. Narrative is evaluative and produce lessons for the future (Hagström and Gustafsson, 2019: 390). In this sense, Trump 2.0’s narrative to his audience of followers seeks to reinstate respect for US strength and its position in the world by articulating a future akin to the one experienced in the remote past, while also preventing the possible future reoccurrence of circumstances existing in the recent past (i.e. under the previous government) (undesirable future). Such a framing and handling of the US master role targets the people versus the elites domestically, as well as, internationally, other states seen to be taking advantage of and disrespecting the country. This narrative on the master role has a domestic political rationale of self-preservation, legitimation of the project and the displacement of political adversaries, while it also indicates to the external audience of competitors renewed strength and direction:

From this day forward, our country will flourish and be respected again all over the world. We will be the envy of every nation, and we will not allow ourselves to be taken advantage of any longer. During every single day of the Trump administration, I will, very simply, put America first […]. America will reclaim its rightful place as the greatest, most powerful, most respected nation on earth, inspiring the awe and admiration of the entire world. (Trump, 2025a)

Trump 2.0 also narrates in an emergent present not only his aspiration for the US to be admired and respected; he also warns other states regarding the disrespect the country has experienced over time, something he will stop by his newly invigorated enactment of the master role towards friends and foes: ‘We have been ripped off for decades by nearly every country on Earth, and we will not let that happen any longer’ (Trump, 2025b). Further, he also adds in relation to China and other states of the international system:

And again, I have great respect for President Xi of China, great respect for China. But they were taking tremendous advantage of us. And I commend them for that. I say, hey, if you can get away with it, that’s OK. But they understand exactly what’s happening and they probably, most of them are saying it’s about time. They did something, and they’re going to fight and they’re going to fight fair. Everyone’s going to fight. I say to the leaders, look, you got to take care of your country, but we have to start taking care of our country now. (Trump, 2025c).

As part of Trump’s account of reinstating admiration and respect for the US master role, he also includes elements of a successful story to show his policies work and create the desired effects. Trump strategically stretches the story to show the value of his policies, which means that the role conception and role enactment of the master role start to be fully aligned and no longer are severely mismatched. In other words, with Trump supposedly having repaired the master role of the US, other countries start to show their admiration anew and thus no longer disrespect said role. For Trump, other states have also started agreeing and complying with the new demands and policies of the US forming part of his quest to revindicate its lost stature. While the binary notions of disrespect/respect are more strongly present when it comes to trade issues, the narrated success of trade policy and how the country is once again admired and respected with regards to its master role, they also permeate the security dimension—including, for instance, in discussions to bring peace to Gaza and Ukraine. In a bilateral encounter with Starmer in Scotland, where Trump also met with President of the EU Commission Ursula von der Leyen to negotiate a trade deal in line with his current tariff policy, the US head of state spoke of trade and security issues alike. He depicted his country as a new international actor due to his unique entrepreneurial leadership skills:

And our country’s, pretty soon, I think I can say it right now, our country’s never done better. We had a country that was dead. It was dead six months ago, a year ago. Leaders from NATO, we had a very successful meeting at NATO. Your Prime Minister was there. It was an unbelievable love fest with all of those countries and they agreed to go [to] 5% and all the things, very smart to do. But many of the leaders, essentially they said, ‘Your country was dead one year ago’. And we did, we had a dead country. We had a president who didn’t know where the hell he was. We had horrible, horrible people running our country. (Trump, 2025d)

This narration of the US as successful actor as a result of it having regained others’ respect for its master role is also to be found in a meeting with ASEAN leaders held in Malaysia, and then later in one with President Xi of China during the APEC Summit taking place in South Korea in October 2025, too. In the first of these meetings, Trump stresses the bond between the US and Asian states, the North American country’s renewed leadership and the type of trade agreements recently achieved or soon to be concluded. Yet, he also highlights how none of this could have been achieved without the US adopting a privileged position, essentially meaning its stature now being recognised and respected by others: ‘The United States is having its Golden Age. Last year we were a country that were doing very poorly under a semi leadership. I do not even call it leadership’ (The White House, 2025a).

In the second meeting, meanwhile, both parties acknowledge each other as great leaders, but Xi speaks of Trump as the figure making American Great again and as a leader who has contributed to world peace in reference to his initiatives in the Middle East and between Russia and Ukraine. In follow up, Trump (2025e) mentions the good results of the bilateral meeting with China when heading back to the US on Air Force One—cited as indicative of how the recent measures adopted by the US are working well and achieving the desired results for the American people (i.e. a new deal with China) (see also, White House, 2025b). On Truth Social, Trump closes his post regarding the trip to Malaysia, South Korea and the set of agreements reached with China by emphasising the new respect felt for the US: ‘Our Nation is Strong, Respected and Admired Again and, THE BEST IS YET TO COME!’ (Trump, 2025f).

Thus, Trump 2.0’s narrative on the US’s master role is about vindicating anew the strength and the leading position of his country in the international system—in other words, it is a status narrative (Bywaters, 2025). The starting point for this is a perceived mismatch or gap between role conception and how the master role was enacted by his immediate predecessor. The master-role narrative has a key domestic dimension to it, then: namely, to seduce and attract followers to the political project (Berenskötter, 2020). Further, the story relies on stressing elements of the country being disrespected by national elites, as embodied by the figure of Biden and his government as well as peer states in the international. This recent past becomes key ‘storical’ material to prevent an undesirable future. Only Trump as the main storyteller can rescue and save the country and reinstate respect for US standing. Moreover, the master role narrative always unfolds in the temporality of an emergent present as Trump goes back to the recent past to provide narrative justification for the current sense of disrespect, striving to offer an alternative to it. This constitutes an acceptable vision of the future, albeit one always based out of times gone by; in this case, a more remote past in the story of the US as a nation (i.e. the heartland). The future is an uncertain landscape in narrative framings, but the constant reference to the heartland of the US developed by Trump seeks to project a degree of certainty when indicating to the people, to his followers, that such a future has been lived before by the country itself and the people’s own ancestors. In other words, Trump relies on the cultural fabric of the US to project a vision of the future driven by a glorious past to his domestic and international audiences in consolidating power.

## Conclusion

This paper has adopted an interpretive approach in line with the analytical benefits of drawing on symbolic interactionist role theory and strategic narrative analysis in the foreign policy realm. It turned also to the notion of the heartland derived from populism studies. These were the respective cornerstones of the theoretical elaborations offered. So doing helped unpack how Trump has narrated the conception and enactment of the master role (or status) of the US within the framework of the story he has cast and made manifest at home and abroad after becoming president for a second term.

The main theoretical contribution advanced hereby is that the master role (status) can be performed and enacted via storytelling; most role-theory accounts have until now assumed the master role to only become active through auxiliary roles or functions such as leader, security provider, mediator and so on. In this interpretive account, the master role has its own life within the story adopted by the narrative entrepreneur. In addition, the gains generated for the scholarship also lie in what actors perceive the master role to be and how it has been performed over time, creating overall consistency or a gap between role conception and role enactment. While this gap has been neglected in studies invoking role theory, what has been shown here is how leaders can stretch and manipulate via strategic narratives the distance between conception and enactment. This helps carve out new space to perform a given role of the state in the international; in this case, the master role of the US.

Moreover, this paper also provided an understanding of populism rooted in the heartland, revealing the key elements of the United States’ master-role narrative under Trump. Only by narrating a future in a retrospective way—that is, connected to a glorious national past (i.e. heartland)—does the future become desirable and indeed attainable in the eyes of the people of the populist project. It is communicated accordingly to international peers. As the future is always inherently unpredictable, by narrating a story of a magnificent past which exists in the collective memory of the American people, Trump is able to provide a certain sense of stability about what comes next. Thus, the story told by narrative entrepreneur Trump is only possible in an emergent present where past, present and future interlock, representing a key way to give direction and a certain level of coherence to what is conveyed.

The strategic narration of the master role of the US has both an international and domestic dimension to it. The story of its international components is about indicating to external peers the need to show more deference and respect to the US, further to being a means by which to justify policy positions and new ways of dealing with friends and foes alike. It is also a key pathway to show the success of US foreign policy in its pursuit of respect under Trump’s leadership. The domestic dimension of status, meanwhile, has typically been neglected in previous accounts despite being equally key. Reference to the US master role has a clear political motivation: it helps achieve concrete goals such as legitimising the populist project and mobilising supporters behind it.

Finally, this paper can be seen as the starting point to engage with the notion of status more broadly and how it has systemic and agential aspects in the international, ones which need further unpacking when studying populist foreign policy. More specifically, whether the case of reinstating lost status is something which is only present in the US given its great power master role needs further investigation on the basis of taking a comparative approach to populist foreign policy from a leader-centric perspective. It is populist leaders who are the central actors in such storytelling, as they have personalised foreign policy—enjoying sufficient room to construct and narrate accounts of respect/disrespect when it comes to the master role in the international. Another element deserving of further investigation is how the populist leader not only tells a story but also acts it out. While examining this latter aspect was not a central goal of this contribution, performative theatrical elements were certainly identified as present in how the story of the US’s master role has been delivered. This theatrical aspect is something yet to receive full scholarly attention—not only as regards populist foreign policy but also IR’s narrative turn.

What this study has done, however, is to show the value of adopting an interpretative framework when analysing the symbolic component of status; in this particular case, role theory and the strategic narrative of US foreign policy. Narratives in their strategic and purposeful sense are where roles and role relationships exist and take form. In the case of the stories curated and advanced by agents of populism, moreover, they are where master roles unfold via a retrospective future claiming to keep the heartland alive.

## Data Availability

No datasets were generated or analysed during the current study.
